# Deactivation of Cu‐Exchanged Automotive‐Emission NH_3_‐SCR Catalysts Elucidated with Nanoscale Resolution Using Scanning Transmission X‐ray Microscopy

**DOI:** 10.1002/anie.201916554

**Published:** 2020-02-28

**Authors:** Xinwei Ye, Joel E. Schmidt, Ru‐Pan Wang, Ilse K. van Ravenhorst, Ramon Oord, Tiehong Chen, Frank de Groot, Florian Meirer, Bert M. Weckhuysen

**Affiliations:** ^1^ School of Materials Science and Engineering Key Laboratory of Advanced Energy Materials Chemistry (MOE) Collaborative Innovation Center of Chemical Science and Engineering (Tianjin) Nankai University Tianjin 300350 P. R. China; ^2^ Inorganic Chemistry and Catalysis Group Debye Institute for Nanomaterials Science Utrecht University Universiteitsweg 99 3584 CG Utrecht Netherlands

**Keywords:** automotive catalysis, catalyst deactivation, copper, X-ray microscopy, zeolites

## Abstract

To gain insight into the underlying mechanisms of catalyst durability for the selective catalytic reduction (SCR) of NO_*x*_ with an ammonia reductant, we employed scanning transmission X‐ray microscopy (STXM) to study Cu‐exchanged zeolites with the CHA and MFI framework structures before and after simulated 135 000‐mile aging. X‐ray absorption near‐edge structure (XANES) measurements were performed at the Al K‐ and Cu L‐edges. The local environment of framework Al, the oxidation state of Cu, and geometric changes were analyzed, showing a multi‐factor‐induced catalytic deactivation. In Cu‐exchanged MFI, a transformation of Cu^II^ to Cu^I^ and Cu_*x*_O_*y*_ was observed. We also found a spatial correlation between extra‐framework Al and deactivated Cu species near the surface of the zeolite as well as a weak positive correlation between the amount of Cu^I^ and tri‐coordinated Al. By inspecting both Al and Cu in fresh and aged Cu‐exchanged zeolites, we conclude that the importance of the preservation of isolated Cu^II^ sites trumps that of Brønsted acid sites for NH_3_‐SCR activity.

## Graphical abstract

**A look at the exhaust**: The deactivation of NO_*x*_‐reduction catalysts based on Cu‐exchanged SSZ‐13 and ZSM‐5 zeolites was studied by scanning transmissionX‐ray microscopy, which enables a spatial analysis of the local environment of both Al and Cu species at nanoscale resolution. The importance of isolated Cu species was shown and a correlation between zeolite lattice destruction and an inactive Cu phase was established.

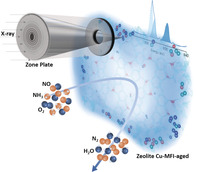

## Introduction

Increased fuel efficiency in diesel vehicles results in the undesirable production of environmentally damaging nitrogen oxides (NO_*x*_).^1^, ^2^ The commonly employed solution in mobile vehicles is ammonia selective catalytic reduction (NH_3_‐SCR) of NO_*x*_ over Cu‐exchanged zeolite catalysts. This was first proposed in a landmark study in 1986 with Cu‐exchanged zeolite ZSM‐5, which showed high activity, but had insufficient lifetime under the harsh conditions of a real vehicle.^3^ To meet the actual requirements of a mobile tailpipe, SCR catalysts must maintain activity throughout the lifetime of a vehicle while experiencing a wide range of temperatures between 150 and 550 °C in the presence of steam as well as hydrocarbons and other poisons (for example, sulfur).^4^, ^5^ Another Cu‐exchanged zeolite, SSZ‐13 (CHA framework), has recently been commercialized for diesel vehicle NH_3_‐SCR as it provides the required catalytic performance and lifetime. Although it was only first disclosed in the mid‐2000s, this material has already been the subject of numerous studies aiming at further improving its performance and lifetime.^5^

To understand the reasons for the high activity and lifetime of Cu‐exchanged zeolites in the NH_3_‐SCR reaction, mechanistic insights about the reaction are needed, which is why the behavior of both the Cu sites and the role of the Brønsted acid sites are topics of extensive investigations.^6^ It is commonly agreed upon that the reaction pathway is conducted by the Cu^II^/Cu^I^ redox cycle under standard NH_3_‐SCR conditions, where the oxidation of Cu^I^ is considered as the rate‐determining step.^7^, ^8^, ^9^ As for Brønsted acid sites, it is proposed that they act as an NH_3_ reservoir for Cu sites, but are not involved in forming reactive species.^10^ Most of our understanding of Cu speciation and location, and acid properties that might affect the NH_3_‐SCR reaction originates from X‐ray diffraction (XRD),^11^ X‐ray absorption spectroscopy (XAS),^8^, ^11^, ^12^, ^13^ ^27^Al MAS NMR spectroscopy,^12^ Fourier‐transform infrared spectroscopy (FT‐IR),^13^ UV/Vis–NIR diffuse reflectance spectroscopy (DRS),^14^ and electron paramagnetic resonance spectroscopy (EPR).^15^ These studies of Al and/or Cu properties have led to a more complete picture of sites contributing to NO_*x*_ reduction, which gives insight into the catalytic mechanism to guide rational catalyst design. However, to further extend the boundaries of our understanding of NH_3_‐SCR deactivation, spatially resolved chemical studies at resolutions below 100 nm, that is, significantly smaller than the size of most single catalyst particles, are required, which allow studying the behavior and correlation between species within one catalyst particle in an intuitive way.

So far, only a few techniques could deliver the necessary sub‐100 nm spatial resolution and high chemical‐information content.^16^ An atom‐probe tomography (APT) study of Cu‐exchanged zeolites probed Al and Cu clustering after aging with single‐atom sensitivity.^17^ For the sake of a comprehensive study of local chemical information, scanning transmission X‐ray microscopy (STXM) in combination with XANES analysis routinely allows chemical imaging at sub‐100 nm spatial resolution, making it a powerful technique to study heterogeneous catalysts in a spatially resolved manner.^18^ From a STXM measurement, a stack of images is obtained which can be regarded as a three‐dimensional data volume. The *x* axis and *y* axis make up the field of view (FOV) of the scanned area, while the *z* axis represents the scanned energy. Therefore, each scanned pixel contains an X‐ray absorption spectrum, in this case, more specifically, one XANES spectrum. The power of X‐ray chemical imaging lies in the abundant chemical information of detailed local environments revealed in a specific FOV, which enables exploitation of the local relationship between different species.

To reinforce our previous studies on Cu‐exchanged zeolites as automotive‐emission catalysts,^17^, ^19^ here we report the application of STXM to study the local chemical environment and distribution of Al and Cu in two distinct NH_3_‐SCR catalysts, namely Cu‐SSZ‐13 and Cu‐ZSM‐5. Both fresh and aged (135 000‐mile simulation^20^) versions of each catalyst material with similar Si/Al ratios and Cu loadings were studied. By comparing the chemical properties of fresh and aged counterparts, the drastic decrease of NH_3_‐SCR performance in the most deactivated Cu‐exchanged zeolite particles could be attributed to the distortion and aggregation of isolated Cu^II^ expedited by the formation of extra‐framework Al species, as revealed by the heterogeneity in the recorded Al and Cu distribution maps. This finding is complemented with conventional characterization methods including XRD, UV/Vis–NIR DRS, FT‐IR spectroscopy with NO as a probe molecule, and ammonia temperature‐programmed desorption (NH_3_‐TPD). The evolution of both Al and Cu species in Cu‐exchanged zeolites caused by the aging procedure is discussed and the general properties of the deactivated NH_3_‐SCR catalysts are pictured in detail. Due to the spatially resolved nature of the STXM data, it was feasible to explore the chemical properties of Al and Cu in different regions within the examined catalyst particle and make statements on the differences between the performances of fresh and aged Cu‐SSZ‐13 and Cu‐ZSM‐5.

## Results and Discussion

### Structure–Performance Relationships

**Figure 1:**
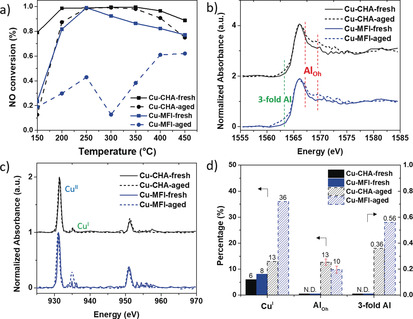
a) NH_3_‐SCR activity of fresh and aged Cu‐exchanged zeolites CHA and MFI. Bulk single‐particle b) Al K‐edge and c) Cu L‐edge XANES of fresh and aged Cu‐exchanged CHA and MFI. Adsorption features of tetrahedral Al (Al_Td_), octahedral Al (Al_Oh_), tri‐coordinate Al (3‐fold Al), Cu^I^, and Cu^II^ are indicated. d) Comparison of the amount of Cu^I^, Al_Oh_, and tri‐coordinate Al in fresh and aged CHA and MFI. The left vertical axis represents the percentage of Cu^I^ and Al_Oh_, the right indicates the integrated pre‐edge area determined from the normalized Al K‐edge XANES. Error bars for the percentage of Al_Oh_ were determined from LSLC (least‐squares linear combination) fitting. Reference information as well as the quantification procedure and results can be found in Figures S2–S4 and Tables S1–S3 in the Supporting Information.

This study was motivated by the NH_3_‐SCR catalytic performance tested on fresh and aged Cu‐exchanged zeolites CHA and MFI. As shown in Figure 1 a, for both fresh Cu‐exchanged zeolites CHA and MFI, a NO conversion of 80–100 % was achieved over the whole temperature regime, though a slow but steady decrease was observed for Cu‐MFI‐fresh starting at a temperature of 250 °C and above. Surprisingly, although the aged Cu‐CHA underwent a steaming process (simulated aging) before starting the reaction, it exhibited high activity and stability for NO conversion, evidenced by the fact that almost 100 % NO conversion was maintained up to a temperature of 350 °C. In contrast, NO conversion was drastically reduced for Cu‐MFI after the aging process, with the highest observed NO conversion being 60 % at the highest temperature (450 °C). These results are consistent with those reported in numerous other studies.^12^, ^21^, ^22^

By comparing two aged zeolites, Cu‐CHA and Cu‐MFI, a significant difference in NH_3_‐SCR activity was observed. One of the most straightforward explanations for this enormous gap in catalytic activity is the differences in stability of the framework structures. However, as indicated by the XRD pattern recorded for our samples, both Cu‐CHA and Cu‐MFI zeolite samples retained their framework structures after the aging process (Figure S1, Supporting Information). On the contrary, it is known that Brønsted‐acidic framework Al atoms can be hydrolyzed or dehydroxylated upon interacting with steam.^23^ Therefore, even though the long‐range order of the zeolite structure might be preserved upon steaming, the local structure of framework Al can change. Subsequently, the nature of Cu ions, which is dependent on the position and density of framework Al, might be affected.

Changes in the local environment of framework Al were evidenced in bulk Al K‐edge XANES. It is a relatively straightforward and convincing way to distinguish and quantify Al coordination as compared to high‐resolution ^27^Al MAS NMR, a commonly used technique that is, however, sensitive to a diminished signal or line broadening especially when paramagnetic species like Cu^II^ are present.^24^, ^25^ The “bulk single‐particle XANES” we discuss is the XANES obtained by averaging all XANES recorded for all pixels of a catalyst particle; details can be found in the Supporting Information. The aging procedure caused a similar effect on Al coordination in both zeolites Cu‐CHA and Cu‐MFI according to Figure 1 b. Although tetrahedral Al, determined by the nature of tetrahedral T sites in the zeolite, was still the dominant species, the emergence of octahedral Al becomes measurable via the occurrence of a shoulder located at the edge position of tetrahedral Al and the peak that becomes visible in the 1569–1575 eV region for both aged zeolites. Furthermore, a pre‐edge feature was observed for the aged zeolites, which was more pronounced in the aged Cu‐MFI zeolite. This notable low‐energy feature in the energy range of 1561–1565 eV was assigned to tri‐coordinated Al, identified by full multiple scattering calculations in a previous study.^25^

A reversible tetrahedral–octahedral transformation of framework Al could be achieved by hydration and dehydration treatment, where either framework Al or Al−OH forms by partial hydrolysis with host water molecules.^26^, ^27^ In this way, Al is saturated with H_2_O, but without loss of lattice Al. In contrast, intensive steaming undoubtedly accelerates the hydrolysis of the framework Al−O bond, especially at high temperatures, directing the irreversible formation of extra‐framework octahedral Al species.^28^ Tri‐coordinated Al was proposed to form by dehydroxylation of Brønsted acid sites followed by dealumination in zeolites, but it was mostly deduced indirectly by combining ^27^Al MAS NMR, EPR, and FT‐IR spectra recorded after steaming or heating.^29^, ^30^, ^31^ The distorted local environment and large quadrupolar coupling constant of tri‐coordinated Al results in NMR invisibility, so a probe molecule was employed to certify their fine structure.^32^, ^33^ The most direct observation of this Al coordination is achieved by XAS via the presence of the aforementioned pre‐edge feature that is indicative of the presence of empty mixed s, d, and p orbitals, that is, the nature of the unsaturated Al.^25^ Previous observations of tri‐coordinated Al were reversible in zeolite H‐Beta and H‐Mordenite, such that it could be restored to tetrahedral Al when exposed to air or wet helium.^25^, ^34^ However, in our case, tri‐coordinated Al was stable in steamed Cu‐exchanged CHA and MFI. This irreversible nature implies that the presence of Cu ions could stabilize tri‐coordinated Al, which was previously considered as a metastable Al species.

Bulk single‐particle Cu L‐edge XANES were collected for all samples using the same setup, where the formal oxidation state of copper, indicated by the edge position, is the most straightforward information obtained. A noticeable absorption at 931.5 eV indicates the predominance of Cu^II^ in all Cu‐exchanged zeolite samples (Figure 1 c). The effect of steaming on Cu‐exchanged CHA was imperceptible. However, in Cu‐MFI, the aging process induced the reduction of Cu^II^ to Cu^I^, evidenced by an obvious increase of the Cu^I^ peak. Cu^I^ sites were also observed in FT‐IR spectra by employing NO as a probe molecule. Apart from the Cu^I^ generated from auto‐reduction of [CuOH]^+^, one additional different Cu^I^ site was detected in aged Cu‐MFI (Figure S5 b). Similar findings of Cu^I^ formation under oxidizing conditions at 400 or 500 °C were previously detected by in situ XANES and Rietveld refinement of in situ powder‐XRD patterns in Cu‐SSZ‐13.^11^, ^13^, ^35^ Additionally, a theoretical study of Cu speciation in CHA indicated that when both H_2_O and O_2_ exist at temperatures higher than 673 K, Cu^I^ located at an unpaired Al site is thermodynamically favorable.^36^ Considering that Cu^I^ has a fully filled d orbital, it is not surprising to observe the formation of Cu^I^, which then is most likely stabilized by defects generated by the steaming of zeolite Cu‐MFI.

**Figure 2:**
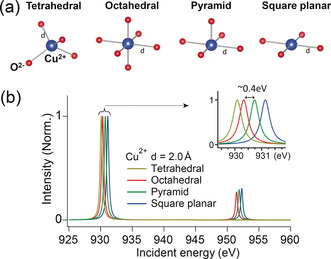
a) Illustration of Cu^II^ in tetrahedral, octahedral, heptahedral (square‐pyramidal), and square‐planar geometries. b) Multiplet calculations of Cu^II^ in different geometric structures with a fixed bond length of 2.0 Å.

Meanwhile, a subtle change of the Cu local environment of Cu‐MFI after aging is evidenced by a slight shift of the Cu^II^ peak in Figure 1 c. The edge position in the Cu L‐edge XANES is an indicator of the Cu^II^ geometric structure due to the crystal‐field splitting of the d orbital.^37^ Although it is difficult to differentiate Cu^II^ coordination geometries in Cu‐exchanged zeolites merely by Cu L‐edge XANES because of the complexity of the Cu^II^ local environment, the shift of the Cu^II^ edge position can be explained by a change of the geometric structure or the Cu−O bond length supported by multiplet calculation (Figures 2 and S6), which was also observed in previous studies.^38^, ^39^ Therefore, during the steaming process in zeolite Cu‐MFI, the Cu^II^ geometry plausibly became distorted from the one of a fully saturated Cu^II^ center. In contrast, no change of the Cu^II^ local environment was detected in Cu‐CHA, explaining its retained NH_3_‐SCR activity.

The bulk XANES spectra provide an overview of catalyst properties that correlate with NH_3_‐SCR performance. Figure 1 d summarizes bulk information obtained from bulk single‐particle XANES of Al and Cu, that is, the relative amounts of Cu^I^, Al_Oh_ (Oh=octahedral), and tri‐coordinate Al of fresh/aged Cu‐exchanged CHA/MFI. There is a clear difference in the Al coordination between fresh and aged NH_3_‐SCR‐active zeolite Cu‐CHA, indicating that part of the tetrahedral framework Al transformed to octahedral Al and tri‐coordinate Al. This dealumination was further evidenced using NH_3_‐TPD by the loss of Brønsted acid sites after aging (Figure S7), indicating that the degree of framework Al degradation was not the dominant factor for catalyst deactivation, especially at temperatures higher than 250 °C, as shown in Figure 1 a. The limited impact of the Brønsted acidity based on tetrahedral framework Al was previously studied both kinetically and spectroscopically, showing that Brønsted acid sites were not the rate‐limiting step in the NH_3_‐SCR reaction cycle and even that extra‐framework Al could have a positive influence on the reaction rate.^40^, ^41^, ^42^

The common feature shared by these three active Cu‐exchanged zeolites is the low percentage of Cu^I^, emphasizing the vital role played by the nature of Cu species in NH_3_‐SCR. The Cu redox cycle is involved in the NH_3_‐SCR reaction where both reduction and oxidation half‐cycles would generate N_2_.^7^, ^43^, ^44^ In Cu‐MFI‐aged, a high percentage of Cu sites started their redox cycle from Cu^I^(NH_3_)_2_ oxidation, which is considered as the rate‐limiting step at temperatures lower than 250 °C, and that Cu^I^(NH_3_)_2_ migrates and is then oxidized to Cu^II^(NH_3_)_2_.^7^, ^8^, ^45^ The local defects generated by steaming might constrain this diffusion of Cu^I^(NH_3_)_2_, which is linked with the low NO conversion in Cu‐MFI‐aged. With increasing temperature, the NH_3_ ligand releases, and the oxidation of Cu^I^ is thermally accelerated and thus the activity is restored in the higher‐temperature regions of NH_3_‐SCR.

In comparison with Cu‐MFI, Cu‐CHA, having a smaller pore size, was more resistant to hydrothermal treatment with less Al leaching, as previously reported.^21^ Therefore, the higher activity and stability of Cu‐CHA‐aged can be attributed to well‐preserved Cu^II^ sites that balance framework Al, in both terms of oxidation state and geometric structure, which enables NH_3_ solvation during reaction. In contrast, the Cu^II^ sites in Cu‐MFI seemed more susceptible to the aging treatment. Apart from the higher percentage of Cu^I^ in aged Cu‐MFI, our bulk single‐particle Cu L‐edge XANES data together with the multiplet calculations provide evidence for a more distorted local structure of Cu^II^.

### Phase Heterogeneities within Catalyst Particles

The scanned area was processed in a spatially resolved manner by principal component analysis (PCA) and *k*‐means clustering after filtering background pixels (more details in the Supporting Information). With this method, the most similar spectra end up in the same cluster, which allows the analysis of the spatial distribution of different phases in a sample without using a priori knowledge about the phases that are present. Here, it should be noted that the clustering result is only based on spectral correlations and not influenced by any spatial correlations. It should also be added that the number of clusters is assigned manually and not an indication for the actual presence of three different spectroscopic phases; *k*‐means clustering will always produce the number of clusters used as an input parameter. In this case, we intentionally over‐clustered the data by using three or four clusters to check for any spectroscopically distinct and possibly minor phases, which would emerge in the average XANES of such (over‐)clustered data. Therefore, clusters are the result of grouping pixels based on the similarity of their spectra.

In zeolite Cu‐CHA‐fresh, a spatially uniform local chemical environment of Al and Cu was found within zeolite particles, indicated by a segmentation result of the measured particles and the corresponding XANES shown in Figure S8. In contrast, in aged Cu‐CHA, a slightly lower amount of tri‐coordinate Al was observed in Al Clus3 and Cu Clus2, which are preferentially present at the edge of the particle and showed a slightly significant increase of Cu^I^. However, the crystal size of zeolite CHA was found to be 100–250 nm by SEM analysis (Figure S9), suggesting that the STXM‐measured particle was an agglomerate of nanocrystals. The spatial resolution of 50 nm (beam size) was not high enough to study differences in the chemical distribution of Al or Cu in a single‐nanocrystal zeolite CHA. Thus, the tiny variation among clusters found in Cu‐CHA cannot be used as an indicator of Al or Cu heterogeneity at the single‐crystallite level but only at the nanocrystal‐agglomerate level.

**Figure 3:**
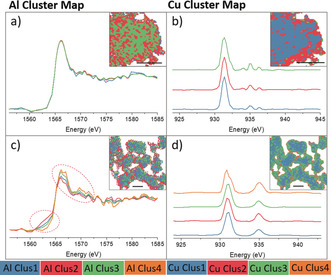
Results of PCA and clustering analysis on the Al and Cu STXM‐XANES of Cu‐MFI. a) Al K‐edge XANES and b) Cu L‐edge XANES and the corresponding distribution map (inset) of Cu‐MFI‐fresh. c) AL K‐edge XANES and d) Cu L‐edge XANES and the corresponding distribution map (inset) of Cu‐MFI‐aged. The intensity of the Cu^II^ L‐edge was normalized to 1 (as done in the processing of the bulk spectra). In the inserted distribution maps, the scale bars represent 1 μm and the pixel size is 25×25 nm^2^. The FOV of the inserted map is 2.15×2.05 μm^2^ in (a,b) and 5.05×5.30 μm^2^ in (c,d).

The crystal size of Cu‐exchanged MFI is 0.2 to a few microns, which enables spatial analysis at the single‐particle level. It is important to emphasize that this zeolite was an industrially manufactured catalyst, making insights especially valuable and difficult to obtain. Figure 3 shows Al and Cu cluster maps of fresh and aged Cu‐MFI. Tetrahedral framework Al is distributed homogeneously in fresh Cu‐MFI. Although the Cu clusters in Cu‐MFI‐fresh exhibited a pattern suggesting that Cu Clus1 was surrounded by Cu Clus2, and Cu Clus3 is preferentially located at the edge of the particle, the dissimilarity of the corresponding XANES spectra was found to be negligible within the noise level (Figure 3 b); the three clusters were therefore interpreted as identical with respect to Cu oxidation state. Within the noise level, we did not find evidence for the presence of more than one spectroscopic phase for both Al and Cu for the single‐catalyst particle data in fresh Cu‐MFI.

As for the deactivated Cu‐MFI‐aged, statistically significant spatial heterogeneities of spectroscopically different phases of Al and Cu were discriminated within a catalyst particle (Figure 3 c,d). A pattern of clusters was found, illustrating the slightly different Al geometries of octahedral and threefold coordination. A strong degree of framework degradation was found to be preferentially located at the external surface of individual zeolite particles, as is evidenced by the distribution of Al Clus2 (representing the phase with tri‐coordinate Al) and Al Clus4 (more octahedral Al) in Figure 3 c. The oxidation state of Cu within this aged zeolite particle was also not completely uniform. The Cu^I^ percentage was different between clusters and a shoulder peak assigned to Cu^II^ was found in Cu Clus4, which was determined to be a hot spot at the edge of the particles (Figure 3 d). The Cu^II^ L‐edge was split into two contributing peaks with an energy gap of around 1 eV, which could be explained by tetrahedral and square‐planar Cu^II^ supported by the measured references (Table S1), multiplet calculations, and literature.^38^ The absolute edge position in Cu L‐edge XANES could not give an accurate coordination number without measuring Cu compounds in well‐defined geometric structures. However, the observed shoulder with an energy gap of 1 eV, together with the main peak in the Cu^II^ edge, presents strong evidence for the co‐existence of two different geometric structures, square‐planar and tetrahedral. We have included this discussion to reinforce the importance of carefully considering what can be considered as “real” differences in these analyses.

### Spatial Correlation between Al‐ and Cu‐Species

**Figure 4:**
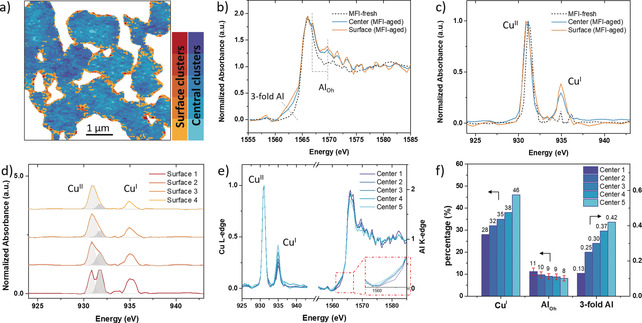
Spatial analysis on individual catalyst particles of aged Cu‐exchanged zeolite MFI. a) Cluster map of both surface (orange) and central (blue) area in Cu‐MFI‐aged, where the color gradient represents a pool of pixels sharing similar spectral features. The classification of pixels was based on the deviation of their Cu L‐edge spectra from the average spectrum of all pixels. Surface clusters 1–4 represent regions with more degraded Al and Cu species, which were predominantly found on the surfaces of individual particles. The central clusters 1–5 mostly describe the bulk. Sum spectrum of b) Al K‐edge XANES and c) Cu L‐edge XANES of surface and central clusters in comparison with the fresh Cu‐MFI. d) Cu L‐edge XANES of surface clusters 1–4 and a deconvolution of the Cu^II^ peak. e) Cu L‐edge XANES and Al K‐edge XANES of central clusters 1–5. f) Relative amounts of Cu^I^, Al_Oh_, and tri‐coordinate Al determined from the spectra in (e). The left vertical axis represents percentage, the right one the integrated area of the pre‐edge region in Al K‐edge XANES.

The square‐planar Cu^II^ in Cu Clus4 (Figure 3 d) implied the formation of Cu_*x*_O_*y*_ nanoparticles,^46^ which are regarded as an inactive species in NH_3_‐SCR.^47^ To investigate their spatial distribution more closely, pixels with an asymmetric Cu^II^ L‐edge peak, such as the one shown in Figure 3 d, that is, Cu^II^ present in multiple geometric structures, were selected and divided into four groups that varied in their fraction of square‐planar Cu^II^. It is clear from Figure 4 a that these pixels with square‐planar Cu^II^ were almost exclusively located at the surface of the individual particles, that is, within a few hundred nanometers of the surface of each catalyst particle. This distribution implies that the aggregation of Cu happened near the particle surface, where it then gradually formed Cu_*x*_O_*y*_ nanoparticles. This partial Cu^II^ agglomeration in Cu‐MFI‐aged was also evidenced in UV/Vis–NIR DRS spectra (Figure S10), showing the additional absorption of Cu_*x*_O_*y*_ at around 40 000 cm^−1^ when compared to its fresh counterpart.^14^ These results strongly indicate that part of Cu^II^ in Cu‐MFI was no longer present as isolated sites after aging.

The deactivation of the Cu‐exchanged MFI has previously been linked to the affinity of Cu to form Cu−Cu and Cu−Al bonds, indicating the formation of Cu_*x*_O_*y*_ nanoparticles or a CuAl_*x*_O_*y*_ spinel phase, respectively.^12^, ^17^, ^48^ By correlating the Al and Cu STXM data we could obtain additional information about the spatial correlation between Al and Cu: the surface area containing a large fraction of Cu_*x*_O_*y*_ and Cu^I^ also shows partial destruction of the zeolite framework (Figures 4 b,c and S11). Due to the co‐occurrence of octahedral Al and tetrahedral Cu, a CuAl_*x*_O_*y*_ spinel phase cannot be excluded in our case. The destroyed surface layer of Cu‐MFI‐aged can be explained by the fact that the surface area of each zeolite particle was the first region exposed to steam and therefore experienced the most severe steaming treatment. The detachment of extra‐framework Al then induces migration and subsequently aggregation of Cu ions because it is no longer necessary to have a cation to balance the negative charge from the framework Al. In turn, the aggregation of freed, isolated Cu ions causes them to lose their charge‐balance ability, which further enhances the severe framework damage in zeolite MFI. The interplay between degraded Al and Cu eventually leads to the zoning of inactive species that might further limit diffusion of reactants and products through the catalyst particle because of the collapse and blockage of zeolite channels on the catalyst‐particle surface. All of this leads to the “perfect storm” for deactivation.

Severe degradation of Al and Cu species was predominately found in the region of around 250 nm from the surface. However, the other areas within particles that were less degraded revealed more general chemical properties of the catalyst. Hence, for a more detailed analysis of these areas, pixels with square‐planer Cu^II^ composite, that is, the surface region marked in orange in Figure 4 a, were filtered before inspecting the main body of each particle of Cu‐MFI‐aged. Thus, Cu L‐edge XANES of the remaining pixels showed a single Cu^II^ L‐edge peak representing distorted tetrahedral Cu^II^ or octahedral Cu^II^. The classification of the central part of the catalyst particle was achieved by examining the first two principal components (PC) based on the fact that PC1 closely resembles the average spectrum of all pixels (after background removal) and PC2 represents the strongest deviation from PC1 (being an orthonormal basis vector to PC1 and capturing the second largest variance in the data). It is clear in Figure S12 that the pixels with a higher contribution of PC2 contained a lower percentage of Cu^I^. The clusters Center 1–5 were labeled such that the Cu^I^ amount increased when going from cluster Center 1 to Center 5. The corresponding Al K‐edge XANES and Cu L‐edge XANES of each cluster are shown in Figure 4 e.

The clusters Center 1–5 in Figure 4 e exhibit a spatially random pattern, suggesting that the transformation of Cu^II^ to Cu^I^ does not have a preferred location or direction within individual zeolite particles. Based on the estimated amount of degraded Al species obtained from least‐squares linear combination (LSLC) fitting, all these clusters have a comparable percentage of octahedral Al but vary in tri‐coordinate Al, as illustrated in the zoomed‐in view of the pre‐edge region in Figure 4 e. The trend of increasing threefold Al coordination when going from cluster Center 1 to Center 5 was accompanied by an increasing Cu^I^ percentage. Undoubtedly, hydrolyzation or hydroxylation of Brønsted‐acidic sites would destroy the framework structure, directing the irreversible formation of defect sites consisting of tri‐coordinate Al, such as Al−OH and AlO^+^.^25^ Subsequently AlOOH, Al(OH)_2_^+^, and Al(OH)_3_ might be formed in the presence of water.^12^, ^49^ On the contrary, Cu^I^ was created by auto‐reduction of either [CuOH]^+^ or Cu^II^. The reduction of Cu^II^ was proposed to be associated with a thermally driven decomposition of H_2_O at temperatures higher than 300 °C, accompanied by the formation of a proton that can balance the framework charge.^50^ However, independent of how Cu^I^ is generated, Cu^I^ could be stabilized by tri‐coordinate Al if it is adjacent to the degraded Al site. Finally, considering the close interaction between leached Al and Cu, the formation of a CuAl_*x*_O_*y*_ phase cannot be excluded by an XAS study,^12^ which was also proposed based on APT studies.^17^, ^19^ We have elucidated the deactivation of Cu‐exchanged zeolites in terms of local environment and the spatial distribution of both Al and Cu. The sensitivity of XAS to the geometric structure enabled us to discover the NMR‐invisible tri‐coordinate Al, which exists in both aged Cu‐CHA and aged Cu‐MFI. Since dealumination was found in the NH_3_‐SCR‐active catalyst Cu‐CHA‐aged, the limited degree of framework destruction was not the main reason for the loss of activity. Instead, the loss of isolated Cu sites, which transformed into Cu_*x*_O_*y*_ nanoparticles or a CuAl_*x*_O_*y*_ spinal phase, and was largely located on the particle surface, is more detrimental to the reaction. It was previously inferred from XPS and calorimetry upon adsorption of the probe molecule NH_3_ that the location of defect sites in zeolite ZSM‐5 is mainly concentrated on the external surface of the zeolite crystals.^51^ By our spatial analysis of the data recorded for zeolite Cu‐MFI‐aged, we could now directly visualize this, revealing that the most degraded framework structure is located at the edges of the individual catalyst particles and goes along with the formation of inactive Cu_*x*_O_*y*_ nanoparticles which further limit the accessibility of less destroyed Cu sites in the central part of the catalyst particles due to framework collapse and channel blockage near the surface. This observed Al and Cu zoning is a good example of nanoscale destruction in industrial catalysts, which is caused by the surface migration of nanoparticles.^52^ The discovery of this minority species of Cu_*x*_O_*y*_ at the surface (4555 surface pixels from a total of 29 759 analyzed pixels) illustrates the power of STXM for nanoscale spatial analysis of catalyst particles.

## Conclusion

The observed variations in catalytic activity and stability between fresh and aged Cu‐exchanged zeolites with different framework structures (CHA and MFI) motivated a detailed study of the deactivation factors with respect to zeolite framework structure, Al species, and Cu species. Therefore, we employed STXM, which combines the main functions of X‐ray absorption spectroscopy (XAS) and high‐resolution imaging, providing abundant information about the local chemical environment in the form of a chemical map of fresh and aged Cu‐exchanged zeolites. Our results indicate that although the degradation of tetrahedral framework Al to octahedral Al or tri‐coordinate Al caused the loss of Brønsted acid sites, NH_3_‐SCR activity was maintained in Cu‐exchanged CHA even after aging, which is attributed to the hydrothermally stable framework structure and well‐preserved Cu^II^. For Cu‐exchanged MFI, the deactivation of NH_3_‐SCR was induced by the evolution of isolated Cu^II^ to both Cu^I^ and Cu_*x*_O_*y*_, resulting in a scarcity of isolated Cu^II^ in aged Cu‐MFI. The spatial distribution of both Al and Cu species was explored by inspecting single‐pixel spectra, revealing spatial correlations between species and information that is lost in bulk data. We observed zoning of extra‐framework Al, Cu^I^, and Cu_*x*_O_*y*_ on the surface of every individual catalyst particle of the most deactivated zeolite, Cu‐MFI‐aged. Then, by investigating the main body of each catalyst particle, that is, excluding the degraded surface regions, a plausible spatial correlation between tri‐coordinate Al and Cu^I^ was established. Overall, we can conclude that the loss of isolated Cu sites is detrimental to NH_3_‐SCR performance. Considering the interdependence between framework Al and isolated Cu species, further investigations of a more durable NH_3_‐SCR catalyst should emphasize the stabilization of isolated Cu active sites. In case of Cu‐exchanged zeolites, preservation of isolated Cu relies on the stability of an Al‐containing framework, that is, a hydrothermally stable zeolite lattice, as a critical factor for NH_3_‐SCR.

## Conflict of interest

The authors declare no conflict of interest.

## Supporting information

As a service to our authors and readers, this journal provides supporting information supplied by the authors. Such materials are peer reviewed and may be re‐organized for online delivery, but are not copy‐edited or typeset. Technical support issues arising from supporting information (other than missing files) should be addressed to the authors.

Supplementary
