# Supplementary material for: Deactivation of Cu‐Exchanged Automotive‐Emission NH3‐SCR Catalysts Elucidated with Nanoscale Resolution Using Scanning Transmission X‐ray Microscopy
Source: Angew Chem Int Ed Engl. 2020 Feb 28;59(36):15610–7. doi: 10.1002/anie.201916554 (PMC7522683; doi:10.1002/anie.201916554)
Supplement: Supplementary file 1 — Supplementary [file ANIE-59-15610-s001.pdf]

Angewandte Chemie
Eine Zeitschrift der Gesellschaft Deutscher Chemiker

Supporting Information

Deactivation of Cu-Exchanged Automotive-Emission NH_3-SCR Catalysts Elucidated with Nanoscale Resolution Using Scanning Transmission X-ray Microscopy

*Xinwei Ye, Joel E. Schmidt, Ru-Pan Wang, Ilse K. van Ravenhorst, Ramon Oord, Tiehong Chen, Frank de Groot, Florian Meirer, and Bert M. Weckhuysen**

anie_201916554_sm_miscellaneous_information.pdf

Supporting Information

Table of Contents

SECTION 1. EXPERIMENTAL PROCEDURES **3**
- Sample Preparation 3
- Characterization Methods 3

SECTION 2. STXM DATA COLLECTION AND ANALYSIS **4**
- Images Alignment, Background Filtration and Normalization 4
- Al K-edge XANES and Cu L-edge XANES 4
- Principal Component Analysis and *k*-means Clustering Analysis 4
- Detailed Analysis of Spatial Information of Cu-MFI-aged 5

SECTION 3. MULTIPLET CALCULATIONS IN A CRYSTAL FIELD OF NEAREST NEIGHBOR **6**

SECTION 4. RESULTS AND DISCUSSION **7**

REFERENCES **15**

AUTHOR CONTRIBUTIONS **15**

Section 1. Experimental Procedures

Sample Preparation

Zeolite SSZ-13 was prepared by combining 25.3 g of 25 wt% solution of a N,N,N-trimethyl-1-adamantammonium (Sachem, pure) with 10 g of a 1 M NaOH solution and 10 g of zeolite Y (Zeolyst, CBV-720), and then water was added to bring the total mass to 70 g. The mixture was homogenized using magnetic stirring, and then divided into several 23 mL Parr autoclaves reactors, placed in an oven at 160 °C and allowed to react for 4 days while rotating. The product was recovered using centrifugation and washed with copious amounts of water and dried in an oven at 80°C. Prior to reaction testing samples were calcined in air by initially holding them at 150 °C for 3 h before heating the samples further to 580 °C for 6 h to remove the organic structure directing compound. After calcination they were exchanged to ammonium form using 1 M NH_4NO_3 (100 mL of solution per gram of catalyst) at 80 °C with stirring for 3 h; this was done a total of three times. NH_4^+-SSZ-13 and NH_4^+-ZSM-5 (Zeolyst, CBV2134) were then calcined to proton form using the standard method in air at 550 °C before ion-exchanged with Cu^{2+}. The Cu ion-exchange procedure was conducted following the literature[1] that 1 g zeolite in proton form was suspended in 250 mL 10 mM Cu(II) acetate solution and stirred at room temperature for 24 h, followed by washing with a large excess of demineralized water. After drying, the catalyst was then calcined again in air using the same calcination program. The calcined Cu-SSZ-13 and Cu-ZSM-5 is labelled as Cu-CHA-fresh and Cu-MFI-fresh respectively in the article.

The aging procedure was to mimic a 135,000 mile vehicle-aged catalyst based on the industry standard.[2] The 10 % steam in air flow was produced by using a bubbler at 47 °C and was first introduced to the fresh zeolite Cu-CHA or Cu-MFI in a porcelain combustion boat in a tube furnace at 120 °C to prevent condensation. The steaming procedure was conducted at 800 °C using a ramping rate of 2°C/min and held for 16 h. The steamed Cu-exchanged zeolites were then cooled down to room temperature in dry air and are labelled as Cu-CHA-aged and Cu-MFI-aged.

NH_3-SCR catalytic testing

The NH_3-SCR activity testing was performed in a plug flow fixed bed reactor as described in our previous study.[1] 50 mg of sieved catalyst (0.425-0.150 mm) was loaded into a 1 cm quartz tubular reactor and firstly calcined with 5 % O_2 in He flow at 550 °C, followed by treating with SCR gas feed (provided by Linde) of 1000 ppm NO, 1000 ppm NH_3 and 5 % O_2 balanced by He under a Gas Hourly Space Velocity (GHSV) of 100,000 h^{-1}. The conversion of NO was calculated when the reaction reached the steady-state and took the average conversion in the stabilization period of 60 min from 150 to 450 °C. The concentration of output gases was analyzed by mass spectroscopy (Hiden Analytical) and gas phase FT-IR spectrometer (PerkinElmer).

Characterization Methods

X-ray diffraction (XRD) patterns of Cu-exchanged zeolites were collected by a Bruker D2 Phaser with a cobalt radiation X-ray source (Co k_α = 1.789 Å). Powdered samples were pressed on the sample holder and rotated at 15 revolutions/min during measurement.

UV-Vis-NIR diffuse reflectance spectroscopy (DRS) spectra were recorded against a pure white reference by a Varian Cary 500 UV-Vis-NIR spectrometer with a DRS accessory. The scanned range of wavenumber was 4000 and 50,000 cm^{-1} with a data interval of 10 cm^{-1} and at a rate of 6000 cm^{-1} min^{-1}.

Inductively coupled plasma-optical emission spectroscopy (ICP-OES) was measured using a SPECTRO CIROSCCD (SPECTRO Analytical Instruments, GmbH–Germany) at GeoLab, Utrecht University. Cu-exchanged zeolites were dissolved at 90 °C overnight by aqua regia with HF solution. After cooling down to room temperature, the solution was neutralized by boric acid followed by dilution to an appropriate concentration.

Morphologies of Cu-exchanged zeolites CHA and MFI were studied on a Philips XL30 Scanning electron microscopy (SEM) at an accelerating voltage of 5 kV or 10 kV.

Temperature-programmed desorption of ammonia (NH_3-TPD) was taken on Micromeritics Autochem II 2920 equipped with a TCD detector. The Cu-exchanged zeolite sample was first degassed in He flow for 1 h at 600 °C with a heating ramp of 10 °C/min. Ammonia then flowed pass the sample at 100 °C, followed by flushing with He at 100 °C for 2 h. The ammonia desorption was tracked using TCD at a temperature ramping rate of 5°C min^{-1} in a 25 mL min^{-1} He flow.

NO-adsorbed Fourier-transform infrared (FTIR) spectra in transmission mode were measured on a Perkin-Elmer 2000 instrument. 20 mg sample was grounded and pressed into a slice to fit the self-supported wafer, which was then placed in a holder with CaF2 window for the FTIR measurement. The sample was dehydrated at 200 °C for 1 h in vacuum of 1.2×10^{-6} mbar, the cell was then cooled down to room temperature. The NO dosing was operated at -188 °C achieved by liquid nitrogen. FTIR spectra was recorded upon NO adsorption at elevated cell pressure from 1×10-1 to 15 mbar by dosing 1 % NO/Ar.

Section 2. STXM Data Collection and Analysis

STXM data Collection

Scanning Transmission X-ray Microscopy (STXM) measurements were performed at the Advanced Light Source (ALS) beamline 11.0.2, Lawrence Berkeley National Laboratory, Berkeley, California, USA. Zeolite samples were dispersed in deionized water followed by drop casting them on a silicon nitride window, which was then mounted perpendicular to the X-ray beam on a piezo motor stage. The chamber was first evacuated, followed by back filling with He to around 0.27 bar to minimize X-ray absorption by air and reduce the drift of the motor stage caused by heat generated during the measurement. The X-ray beam was focused by a 45 nm Fresnel zone plate and sequentially an order sorting aperture with a 90 μm pinhole to a spot size of 50×50 nm^2. A stack of transmission images at various energy points across the Al K and Cu-L edges were collected by raster scanning the sample with a step size of 50 nm.

Images Alignment, Background Filtration and Normalization

STXM data was processed in aXis2000, TXM-Wizard,[3] Athena and Matlab. TXM-Wizard provides various functions for synchrotron-based X-ray microscopy, which are used to get spatially resolved chemical information by performing principle component analysis (PCA) and clustering. The aligned images representing optical density were obtained from processing in aXis2000 and then imported to TXM-Wizard, followed by cropping them where necessary to remove empty pixels. A pixel size of 25×25 nm^2 was generated from the original pixel size of 50×50 nm^2 by oversampling using a factor of 2 using bicubic interpolation to a have spatial exploration of fine differences within catalyst particles. Then the background was determined and filtered based on the X-ray absorption spectrum of each pixel, leaving only the particle of interest in the field of view (FOV) for further analysis.

Background pixels and pixels with extremely noisy XANES (*i.e.* pixels with XANES that could not normalized) were first filtered and the XANES of the remaining pixels were normalized before performing PCA and *k*-means clustering. PCA and clustering were used to group single pixel XANES based on their spectral similarity. An edge jump filter was employed to remove the noisy pixels based on the difference between pre-edge and post-edge, followed by using a normalization filter to remove pixels with a distorted pre-edge or post-edge. After applying an edge jump filter and normalization filter, the X-ray adsorption near edge structure (XANES) of the whole image was obtained by summing up XANES of every pixel. Thus, the bulk XANES represents the overall chemical information of Al or Cu of the catalyst.

Al K-edge XANES and Cu L-edge XANES

The Al K-edge XANES was analyzed in Athena. The spectrum was first normalized before any other further analysis. The least-square linear combination (LSLC) fitting was used to quantify the amount of octahedral Al with the fitting range of 1560.35~1574.35 eV, where ZSM-5 and α-Al_2O_3 were chosen as the references which were measured previously by our group.[4] Because the edges of tetrahedral Al and tri-coordinated Al overlap, so the percentage of tetrahedral Al obtained from LSLC fitting was contributed from both of these Al species. The estimation of the pre-edge feature assigned to tri-coordinated Al was conducted in two ways. The difference spectrum between the aged Cu-exchanged zeolite and its fresh counterpart was used to calculate the area of the pre-edge feature. The area from 1560.35 to 1565.15 eV was integrated. Another way is fitting the shape of edge by an error function followed by fitting the pre-edge region by a pseudo-Voigt function. These two methods gave the same results (Figure S3).

Cu L-edge XANES were extracted from stack of absorption images in TXM-Wizard and the background was subtracted in Origin 9.0 followed by normalizing the maximum absorption to 1 in order to compare the differences between spectra. Since both the L_2 and L_3 edge of the L-edge XANES provide the same information, the L_3 edge exhibiting stronger absorbance was chosen to investigate the Cu species. The peak position of Cu L_3 edge was determined by fitting the peak maximum with Gaussian-Lorentz peak. Cu L-edge XANES presents the intrinsic property of the ground-state configuration of the Cu atom which determines the possibility and accessibility of an electronic transition from Cu 2p to higher unoccupied band. L-edge absorption in Cu^{II} with an unoccupied 3d orbital, the strong absorption at 931~932 eV (L_3 edge), dominates in spectrum followed by a weaker adsorption with ~20 eV energy gap (L_2 edge), referring to the 2p$\rightarrow$3d transition. For Cu^{I} with fully filled d orbitals, the L_3 edge adsorption could be explained by the transition from $2p^63d^{10}$ to $2p^53d^{10}4s^1$ transition[5] and hybridization of 3d-4s orbitals, resulting in a higher L-edge peak energy position for Cu^{I} compared to Cu^{II}.[6] Therefore, the L-edge peak area is related to the hole density in a destination orbital in a Cu atom, implying that the ratio of the integrated peak area reflects the relative amount of the different species. To estimate Cu L-edge peak area, reference spectra of CuO and Cu_2O were normalized to an edge jump of 1.0 at 1000 eV,[7,8] yielding Area(Cu^{II})/Area(Cu^{I}) a factor of 1/1.9, which was then applied to samples.

Principal Component Analysis and *k*-means Clustering Analysis

In order to classify all pixels into different groups according to their spectral features, principal component analysis (PCA) and cluster analysis (CA) were performed after applying the edge jump filter and normalization filter. The pixels remaining after filtering

(each containing a XANES with 80 energy points across the Al K-edge XANES) gave enough signal to obtain clear spectral features, including edge position and edge jump. Based on the eigenspectra and scree plot (cumulative variance explained, CVE), the 80 dimensions (for the Al stack) in each pixel were reduced to two or three dimensions that ensured that 90% of data's variance was captured. The selected two or three PCs were then used to plot the data in two/three-dimensional principal component space (score plot) in which k-means clustering was performed using a user-defined number clusters decided upon inspection of the score plot. Based on the initial result from k-mean clustering analysis, the clusters were then optimized by an Expectation Maximization (EM) algorithm for Gaussian Mixture Modeling (GMM) considering point density. This resulted in a class-membership value for each pixel based on its XANES; this value represents the degree (weight) to which each pixel belongs to a certain cluster. After clustering analysis, the XANES of the different clusters was obtained as the weighted average of the XANE of all points of each cluster and the analysis of cluster XANES was the same as done for bulk XANES. PCA and clustering analysis of the Cu stack was performed in a similar way as for the Al stack. Because the Al scan and Cu scan were obtained separately, the FOV of both scans of the same sample were different.

Detailed analysis of spatial information of Cu-MFI-aged

Due to low absorption at the Al K-edge in each pixel that was only 25 nm after oversampling, it was more promising to start analysis from a Cu scan with a much higher absorption in the Cu L-edge range. All the following processing procedure was conducted in Matlab.

Pixels with square planar Cu^{II} were first selected from the analyzed area. Based on the PCA and clustering analysis performed on the Cu map of zeolite Cu-MFI-aged, Cu L-edge XANES of Cu Clus2 shows two peaks with an energy gap of ~1 eV (Figure 4). This is a sign of the existence of Cu^{II} with both tetrahedral and square planar geometric structures. The slope of two energy points around the shoulder in the Cu L_3-edge region was defined to distinguish pixels with a shoulder as indicated in Figure S13a. A map of the slope was obtained after calculating the defined slope in each pixel shown in Figure S13b. Ranges of the slope were set to group pixels with similarly defined slope. In this way, all pixels were grouped into four clusters (Surface 1-4 in Figure 4), exhibiting different fractions of square planer Cu^{II} indicated by the Cu L-edge XANES. Each cluster was saved as a binary image and then employed on the Al scan as a mask. Hence, both Cu L-edge XANES and Al K-edge XANES of the same cluster could be obtained by employing masks on the Cu stack and Al stack, respectively, and shown in Figure 4 and Figure S11.

The extremely degraded region of Cu-MFI-aged was excluded when studying the evolution of Al and Cu species in the aging process. The most important feature from the Cu L-edge XANES is the area ratio of the Cu^{I} and Cu^{II} L_3-edge. Considering the high noise level of single pixel XANES, it is difficult to examine the peak area of Cu^{I} and Cu^{II} correctly. Therefore, PCA, a statistical method, was applied to inspect the distribution of Cu properties. As seen from the eigenspectra of the first two principal components (PC) in Figure S12a, PC1 resembles the average spectrum of the analyzed pixels and the contribution of PC2 reflects the amount of Cu^{II}. The positive value in the eigenimage of PC2 (Figure S12b) indicates pixels with a higher amount of Cu^{II} compared to the average spectrum, while negative value indicates pixels with a lower amount of Cu^{II}, i.e. higher Cu^{I}. Contribution of PC2 in every pixel varies from around 0.05 to -0.05. By evenly splitting the contribution of PC2 (add marker in Figure S12) into five regions as illustrated in Figure S12, five clusters (Center 1-5 in Figure 4) with different levels of Cu^{II} contribution were determined. Then, both Cu L-edge XANES and Al K-edge XANES of the cluster Center 1-5 were obtained, which is shown in Figure 4e.

The quantification of clusters was conducted in the same way as the 'bulk XANES' and the results are shown in Figure 4f, Table S4, S5.

Section 3. Multiplet Calculations in a Crystal Field of Nearest Neighbor

The edge position reflects the geometric structure of Cu^{II}. The crystal field splitting of the 3d orbital changes the energy level of the d orbital, resulting in different energy gaps between the 4s and 3d orbitals and thus changes the peak positions in the Cu L-edge XANES. In order to learn how the coordinate structure influences the Cu $L_{2,3}$ edge absorption spectra, we employ a multiplet computation program MultiX which constructs the crystal field via the positions and charges of surrounding atoms instead of via the symmetrized matrix elements.[9] The atomic wave function was used in solving the multiplet energy levels, where the multiplet levels were calculated by determining the electron-electron, spin-orbit interactions, and the effect of the crystal-field. In the current model, the crystal environment is approximated to be the superposition of point charges potential of the nearest neighbors, that is the crystal environment was determined only by the position of the nearest neighbor. Four coordinate structures, tetrahedral, octahedral, pyramid, and square plane are presented in Figure 2 and Figure S6 to show the calculated Cu L-edge absorption spectra. All the calculated spectra were normalized to the L_3 feature (~930 eV).

Section 4. Results and Discussion

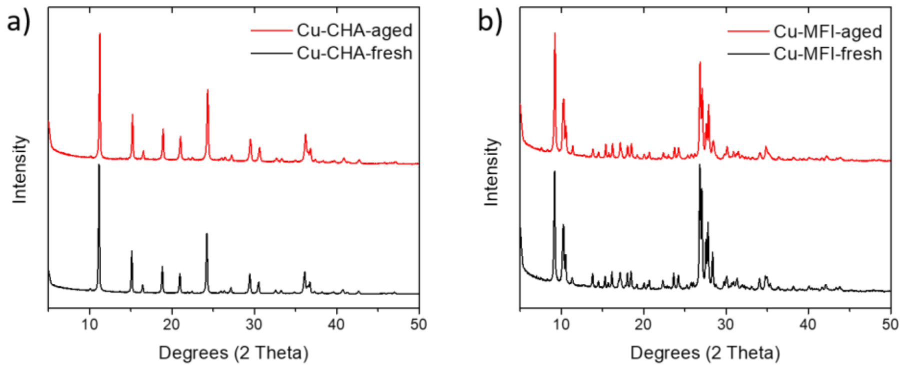

Figure S1. XRD patterns of fresh and aged Cu-exchanged a) CHA and b) MFI. Both Cu-CHA and Cu-MFI maintained their framework structures after the aging process.

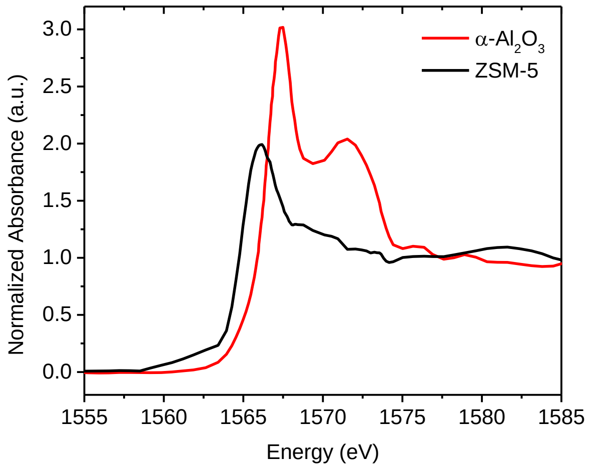

Figure S2. Al K-edge XANES of references (ZSM-5: tetrahedral Al, α-Al_2O_3: octahedral Al). Data was previously collected by our group. The edge positions of the Al K-edge XANES of tetrahedral and octahedral Al exhibit a shift of ~1.3 eV between each other. Furthermore, because of the higher geometric symmetry of the octahedral structure, the XANES of octahedral Al – represented by α-Al_2O_3 – also shows stronger absorption in the near edge region accompanied by a double peak structure with a peak separation of about 4 eV. These distinct spectral features are therefore commonly used to distinguish tetrahedral Al and octahedral Al.

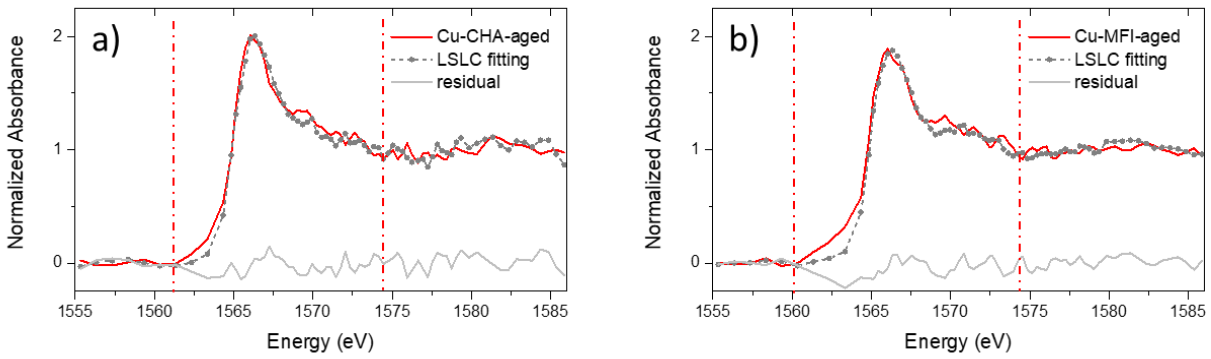

Figure S3. LSLC fitting of Al K-edge XANES of a) Cu-CHA-aged and b) Cu-MFI-aged. ZSM-5 and α-Al_2O_3 were used as references. Fitting range: 1560.35 to 1574.35 eV indicated by red dot-dash lines. Results showed that the Cu-CHA-aged and Cu-MFI-aged had a comparable amount of octahedral Al.

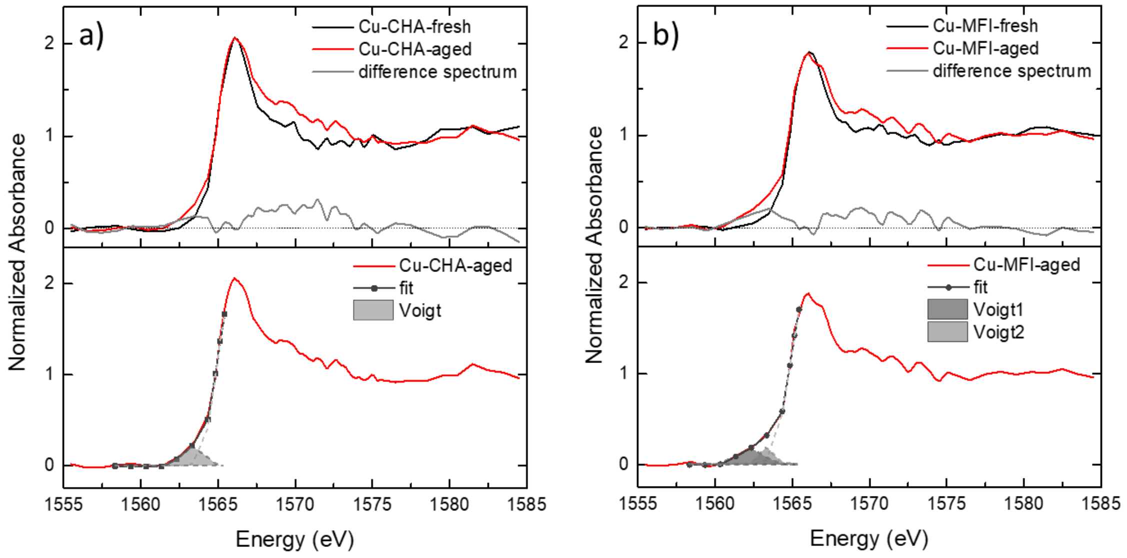

c)	Integral residual area (1560.35-1565.15 eV)	Pre-edge fitting peak area (Voigt function)
Cu-CHA-aged	0.36	0.39
Cu-MFI-aged	0.56	0.37 + 0.20

Figure S4. Estimation of the pre-edge area in a) Cu-CHA-aged and b) Cu-MFI-aged in Al K-edge XANES. The upper panels in a) and b) show the difference spectrum between the fresh Cu-exchanged zeolite and its aged counterpart. The residual area from 1560.35 to 1565.15 eV was integrated. The lower panels in a) and b) show the fitting of the pre-edge area by a Pseudo-Voigt function. The edge was fitted by error function represented by the light grey dash line. The integral area calculated by the above manners is shown in c).

The quantitative analysis of the Al K-edge XANES is shown in Table S2. The percentage of octahedral Al was obtained by least-square linear combination (LSLC) fitting with references for tetrahedral and octahedral Al. Tri-coordinated Al was not evaluated by the LSLC fitting since no appropriate reference exists. While the fitting of octahedral Al relies on both the intense whiteline at 1567.4 eV and the post-whiteline peaks in the spectrum (Figures 1 and S3). The pre-edge region is the only feature that can be used to assess the presence of tri-coordinated Al. Here two methods for estimating the integral area of the pre-edge region were employed, which provided almost identical results (Figure S4). In absence of a reference, the obtained integral pre-edge area was then used to represent and compare the relative amount of tri-coordinated Al in different Cu-exchanged zeolites. Only the aged Cu-exchanged zeolites showed the presence of tri-coordinated Al that the aged Cu-MFI zeolite contained more tri-coordinated Al.

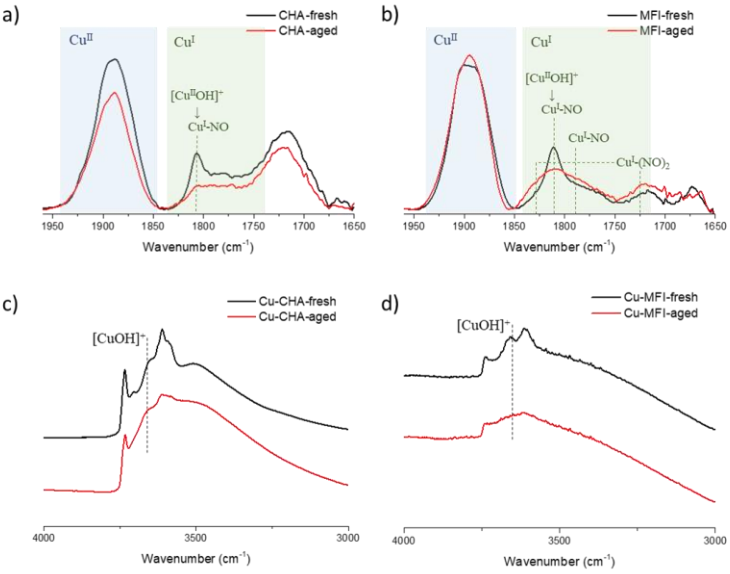

Figure S5. Low temperature NO-adsorbed FT-IR spectroscopy data on fresh and aged Cu-exchanged zeolite in the N-O stretching region of a) CHA and b) MFI at low NO equilibrium pressure of 0.1 mbar at 77 K. The blue label represents mono-nitrosyl adducts in Cu^{II} sites, and the green labels represent the mono-/di-nitrosyl adducts in Cu^{I} sites. FTIR spectra of fresh and aged Cu-exchanged zeolite c) CHA and d) MFI after dehydration in 200 °C for 2h. The indicated band of $[CuOH]^{+}$ is observed in Cu-CHA-fresh/aged (3654 cm^{-1}) and fresh MFI (3658 cm^{-1}). At the same time, the Si-OH, Al-OH and Al-O(H)-Si also could be observed in fresh/aged-CHA and fresh Cu-MFI according to the assignment of the OH group.[10]

The redox ability of the Cu species in the zeolite is pivotal in NH_3-SCR, where $[CuOH]^{+}$ is regarded to have a higher redox ability than bare Cu^{II}.[11,12] As indicated by the result obtained from NO-adsorbed FTIR reported in Figure S5, the fresh Cu-exchanged zeolites CHA and MFI show a well-defined adsorption of the Cu^{I}-NO adduct (bands at 1806 cm^{-1} and 1811 cm^{-1}, respectively) in very low NO coverage under 0.1 mbar. The Cu^{I} detected in the fresh zeolites was generated by auto-reduction of $[CuOH]^{+}$ during dehydration under vacuum at 200 °C for 2h.[13,14] The existence of $[CuOH]^{+}$ in our NH_3-SCR active zeolites Cu-CHA-fresh/aged and Cu-MFI-fresh was also evidenced by the OH stretching band appearing after dehydration in the IR spectra shown in Figure S5. Except for the Cu^{I} generated from auto-reduction, a shoulder at 1785 cm^{-1} tentatively assigned to NO adsorbed on another Cu^{I} site is observed in zeolite Cu-MFI-aged, implying that this Cu^{I} site was originally from Cu-MFI-aged. The abundance of defects revealed by -OH vibration region that hydroxyl group was not resolved any more compared to the fresh counterpart, which might be beneficial to stabilize Cu^{I} in the zeolite Cu-MFI-aged.

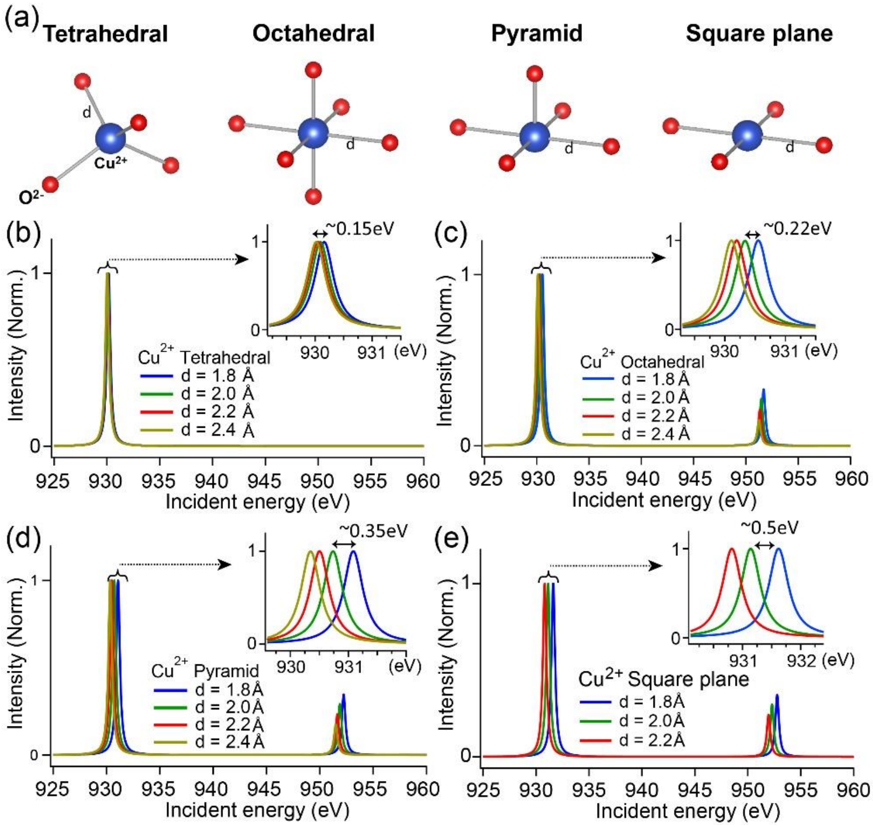

Figure S6. a) Illustration of Cu[II] in tetrahedral, octahedral, square-pyramidal and square planar geometries. Multiplet calculations of Cu[II] in geometric structures of b) tetrahedral, c) octahedral, d) square-pyramid and e) square planar with different bonding length from 1.8 to 2.4 Å, showing a general trend of positive shift of Cu L-edge when shortening Cu-O bond.

Our multiplet calculations of Cu[II] indicate that lengthening Cu-O bonding length causes negative shift of Cu[II] L edge. In order to examine the effect of changing Cu-O bonding length, we studied geometric structures of Cu[II] references and their corresponding L-edge XANES based on previous study.[6] Malachite (octahedral Cu[II]) with a 0.147 Å longer average Cu-O bonding length compared to La_2CuO_4 (octahedral Cu[II]), showed a negative shift of 0.64 eV in Cu[II] L-edge. Similarly, $Y_2Cu_2O_5$ (square planar Cu[II]) with a 0.034 Å longer average Cu-O bonding length compared to CuO (square planar), showed a negative shift of 0.21 eV in Cu[II] L-edge. That is to say, every 0.02 Å longer of Cu-O bond leads to 0.1 eV negative shift of Cu[II] L-edge. In Cu-exchanged zeolites, the average Cu-O bonding length is 1.95 Å. However, even upon steaming treatment up to 750 °C. the average Cu-O bonding length was only 0.02 Å longer analyzed by EXAFS.[15] Therefore, in our case, the splitting of Cu[II] L-edge peak with gap of 1.1 eV would be more reasonable to ascribe to the formation of a Cu[II] phase with a different geometric structure.

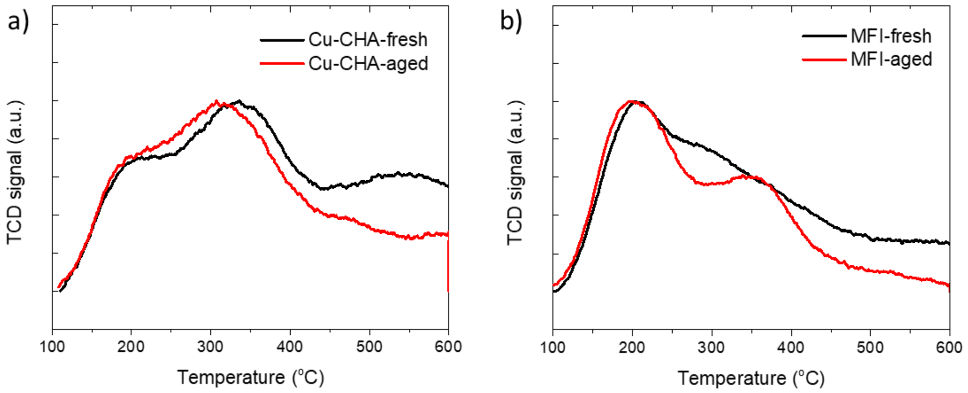

Figure S7. Temperature Programmed Desorption (TPD) of NH_3 of fresh and aged Cu-exchanged a) CHA and b) MFI. The high desorption peak at 500-600 °C is assigned to Brønsted acid sites, which decreased in the aged sample for both CHA and MFI.

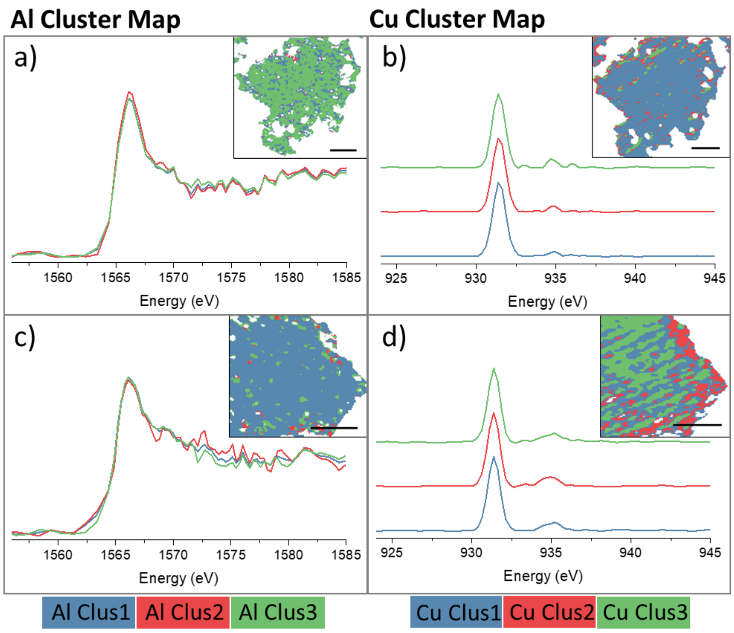

Figure S8. Results of PCA and CA on the Al and Cu STXM-XANES of Cu-exchanged zeolite CHA. a) Al K-edge XANES and b) Cu L-edge XANES and the corresponding distribution map (inset) of Cu-CHA-fresh. c) Al K-edge XANES and d) Cu L-edge XANES and the corresponding distribution map (inset) of Cu-CHA-aged. The intensity of the Cu^{II} L_3-edge was normalized to 1 (done in the processing of the bulk spectra). In the inserted distribution maps, the scale bars represent 1 µm and the pixel size is 25 × 25 nm^2. The FOV of the inserted map is 4.55 × 5.00 μm^2 in a-b), 2.70 × 3.05 μm^2 in c-d). XANES of clusters in each sample are the same within noise level, indicating the homogeneous distribution of Al and Cu species in fresh and aged Cu-CHA.

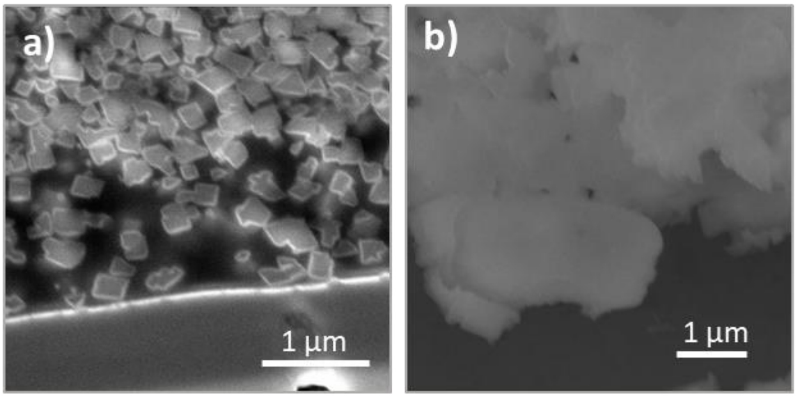

Figure S9. SEM images of Cu-exchanged zeolite a) CHA and b) MFI.

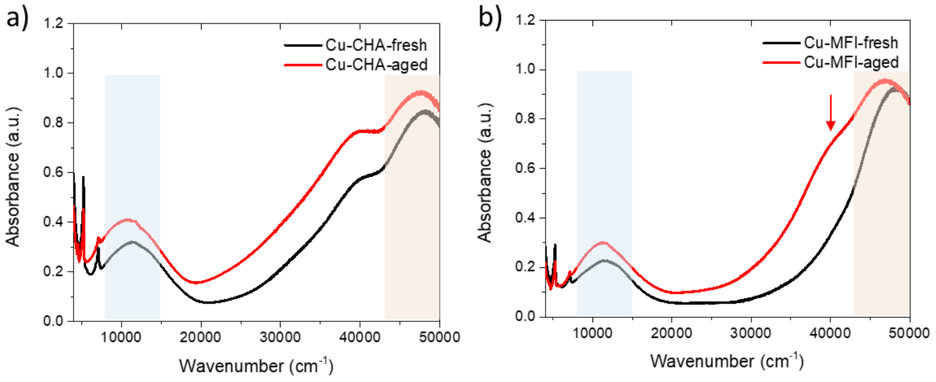

Figure S10. UV-Vis-NIR DRS data of the air-exposed fresh (black) and aged (red) Cu-exchanged zeolites CHA and MFI. The blue and orange shade represent d-d transition in hydrated Cu^{2+} sample and ligand-metal charge transfer (LMCT, $O^{2-}Cu^{2+} \rightarrow O^{-}Cu^{+}$) respectively. The red arrows attribute to the Cu_xO_y species, which formed after steaming in Cu-MFI.

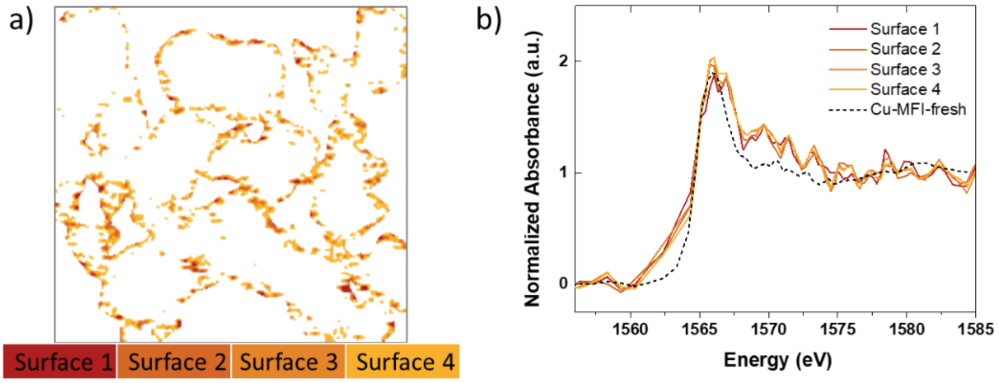

Figure S11. Spatial analysis of the most degraded region in the individual catalyst particles of Cu-MFI-aged. a) Distribution map of the surface clusters labeled as Surfaces 1-4. (b) Corresponding Al K-edge XANES of the Surface clusters 1-4 with a comparison of bulk XANES of Cu-MFI-fresh.

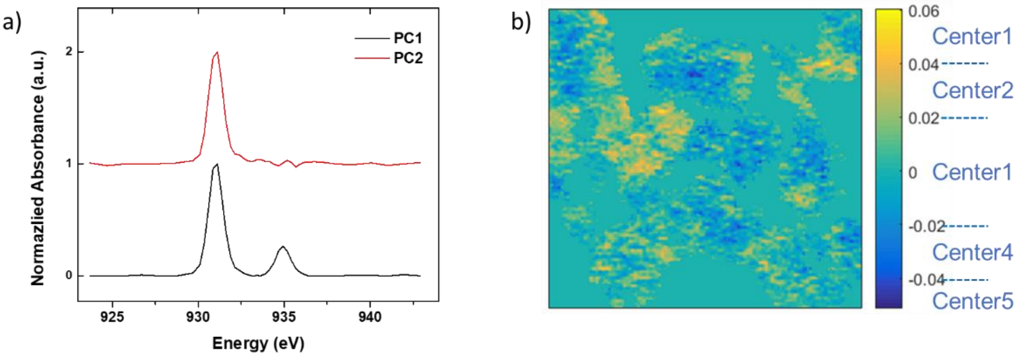

Figure S12. a) Eigenspectra of first two principal components obtained by employing PCA on Cu STXM XANES in zeolite Cu-MFI-aged. b) Eigenimage of the second component (PC2) indicated in Figure S12a and the thresholds setting for clusters Center 1-5.

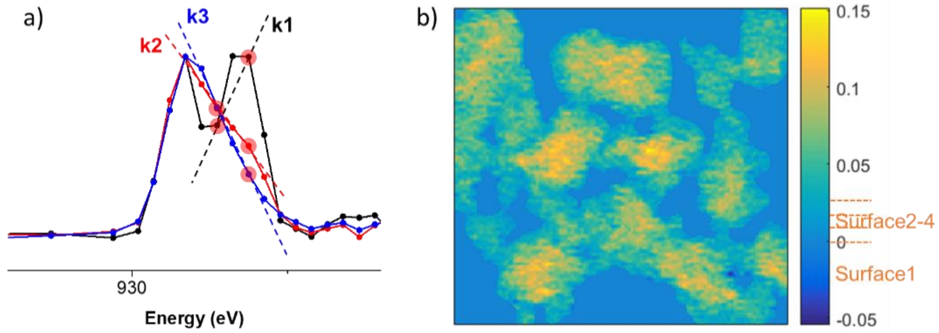

Figure S13. a) The slopes determined from the data points at energy of 931.365 eV and 931.875 eV to discriminate Cu L-edge spectrum with more than one Cu^{II} species (k1, k2). Details are described in the Supporting Information Section 2. b) Map of the slopes (determined in Figure S13a) in zeolite Cu-MFI-aged and the thresholds setting for clusters Surface 1-4.

Table S1. Cu L_3-edge peak information of Cu references measured by STXM.

	L_3 edge position/eV	Oxidation state of Cu	Coordination
CuO	932.04 ± 0.10	+2	Square planar
Malachite	931.65	+2	octahedral
$CuAl_2O_4$	930.93, 931.54	+2	Tetrahedral + octahedral
Cu_2O	934.72	+1	-

Table S2. Quantitative analysis of Al species in fresh and aged Cu-exchanged zeolites.

	(Al_{Td} + 3-fold Al)/%[a]	Al_{Oh}/%[a]	Integral pre-edge area[b]
Cu-CHA-fresh	100	0	N.D.
Cu-MFI-fresh	100	0	N.D.
Cu-CHA-aged	87.3±1.2	12.7±1.2	0.36
Cu-MFI-aged	90.2±1.4	9.8±1.4	0.56

[a] Determined by LSLC fitting using ZSM-5 and α-Al_2O_3 as tetrahedral and octahedral Al references, respectively, and a fitting range 1560.35 to 1574.35 eV.

[b] Integral of the pre-edge region from 1560.35-1565.15 eV.

Table S3. Cu L_3-edge peak information of Cu-exchanged zeolites CHA and MFI.

	Cu^{II} L_3 edge/eV	Cu^{I} L_3 edge/eV	Cu^{I}/%
Cu-CHA-fresh	931.5	935.2	6
Cu-CHA-aged	931.4	935.1	13
Cu-MFI-fresh	931.2	934.9	8
Cu-MFI-aged	931.0	935.0	36

Table S4. Relative amount of Al_{Oh}, tri-coordinated Al and Cu^{I} of clusters Surface 1-4 in zeolite Cu-MFI-aged.

	Al_{Oh}/%[a]	Integral pre-edge area[b]	Cu^{I}/%[c]
Surface 1	12±2.3	1.14	49
Surface 2	12.1±2.2	1.02	45
Surface 3	10.7±2.2	1.00	41
Surface 4	14.6±2.2	0.73	35

[a] Determined by LSLC fitting using ZSM-5 and α-Al_2O_3 as tetrahedral and octahedral Al references, respectively, and a fitting range 1560.35 to 1574.35 eV.
[b] Integral of the pre-edge region from 1560.35-1565.15 eV.
[c] Determined by peak area of Cu^{II} and Cu^{I} edge from Cu L-edge XANES.

Table S5. Relative amount of Al_{Oh}, tri-coordinated Al and Cu^{I} of clusters Center 1-5 in zeolite Cu-MFI-aged.

	Al_{Oh}/%[a]	Integral pre-edge area[b]	Cu^{I}/%[c]
Center 1	11.2±1.6	0.13	31
Center 2	9.7±1.3	0.25	34
Center 3	9.0±1.3	0.3	39
Center 4	8.8±1.3	0.37	42
Center 5	8.1±1.2	0.42	47

[a] Determined by LSLC fitting using ZSM-5 and α-Al_2O_3 as tetrahedral and octahedral Al references, respectively, and a fitting range 1560.35 to 1574.35 eV.
[b] Integral of the pre-edge region from 1560.35-1565.15 eV.
[c] Determined by peak area of Cu^{II} and Cu^{I} edge from Cu L-edge XANES.

References

[1] J. E. Schmidt, R. Oord, W. Guo, J. D. Poplawsky, B. M. Weckhuysen, *Nat. Commun.* **2017**, *8*, 1666.

[2] S. J. Schmieg, S. H. Oh, C. H. Kim, D. B. Brown, J. H. Lee, C. H. F. Peden, D. H. Kim, *Catal. Today* **2012**, *184*, 252–261.

[3] Y. Liu, F. Meirer, P. A. Williams, J. Wang, J. C. Andrews, P. Pianetta, *J. Synchrotron Radiat.* **2012**, *19*, 281–287.

[4] S. Kalirai, P. P. Paalanen, J. Wang, F. Meirer, B. M. Weckhuysen, *Angew. Chem. Int. Ed.* **2016**, *55*, 11134–11138.

[5] S. J. George, S. P. Cramer, M. D. Lowery, E. I. Solomon, *J. Am. Chem. Soc.* **1993**, *115*, 2968–2969.

[6] M. Grioni, J. B. Goedkoop, R. Schoorl, F. M. F. de Groot, J. C. Fuggle, F. Schäfers, E. E. Koch, G. Rossi, J.-M. Esteva, R. C. Karnatak, *Phys. Rev. B* **1989**, *39*, 1541–1545.

[7] R. Sarangi, N. Aboelella, K. Fujisawa, W. B. Tolman, B. Hedman, K. O. Hodgson, E. I. Solomon, *J. Am. Chem. Soc.* **2006**, *128*, 8286–8296.

[8] I. Garcia-Torregrosa, Y. G. Geertzema, A. S. M. Ismail, T. Lee, F. M. F. Groot, B. M. Weckhuysen, *ChemPhotoChem* **2019**, *3*, 1238–1245.

[9] A. Uldry, F. Vernay, B. Delley, *Phys. Rev. B* **2012**, *85*, 125133.

[10] F. Gao, N. M. Washton, Y. Wang, M. Kollár, J. Szanyi, C. H. F. Peden, *J. Catal.* **2015**, *331*, 25–38.

[11] A. Martini, E. Borfecchia, K. A. Lomachenko, I. A. Pankin, C. Negri, G. Berlier, P. Beato, H. Falsig, S. Bordiga, C. Lamberti, *Chem. Sci.* **2017**, *8*, 6836–6851.

[12] C. W. Andersen, E. Borfecchia, M. Bremholm, M. R. V. Jørgensen, P. N. R. Vennestrøm, C. Lamberti, L. F. Lundegaard, B. B. Iversen, *Angew. Chem. Int. Ed.* **2017**, *56*, 10367–10372.

[13] V. L. Sushkevich, J. A. Van Bokhoven, *Chem. Commun.* **2018**, *54*, 7447–7450.

[14] V. L. Sushkevich, A. V. Smirnov, J. A. Van Bokhoven, *J. Phys. Chem. C* **2019**, *123*, 9926–9934.

[15] P. N. R. Vennestrøm, T. V. W. Janssens, A. Kustov, M. Grill, A. Puig-Molina, L. F. Lundegaard, R. R. Tiruvalam, P. Concepción, A. Corma, *J. Catal.* **2014**, *309*, 477–490.

Author Contributions

X. Ye, J.E. Schmidt, I.K. van Ravenhorst, Ramon Oord participated in data acquisition. X. Ye and F. Meirer worked on STXM data processing. R. Wang and F. de Groot worked on the multiplet calculations. X. Ye, T. Chen, F. Meirer and B.M. Weckhuysen discussed data analysis and intepretation. X. Ye wrote the original draft and J.E. Schmidt, F. Meirer and B.M. Weckhuysen contributed to the revisions of the article. B. M. Weckhuysen is the project leader and conceived together with J.E. Schmidt and X. Ye the research plans leading to this work.
